# The solvability conditions for the inverse eigenvalue problem of normal skew *J*-Hamiltonian matrices

**DOI:** 10.1186/s13660-018-1667-1

**Published:** 2018-04-04

**Authors:** Jia Zhao, Jieming Zhang

**Affiliations:** Basic Course Department, Business College of Shanxi University, Taiyuan, P.R. China

**Keywords:** 65F18, 15A51, 15A18, 15A12, Inverse eigenvalue problem, Hamiltonian matrix, Normal matrix, Moore–Penrose generalized inverse, Generalized singular value decomposition

## Abstract

Let $J \in{\mathbb{R}}^{n\times n}$ be a normal matrix such that $J^{2}=-I_{n}$, where $I_{n}$ is an *n*-by-*n* identity matrix. In (S. Gigola, L. Lebtahi, N. Thome in Appl. Math. Lett. 48:36–40, 2015) it was introduced that a matrix $A \in{\mathbb {C}}^{n\times n}$ is referred to as normal *J*-Hamiltonian if and only if ${(AJ)}^{*}=AJ$ and $AA^{*}=A^{*}A$. Furthermore, the necessary and sufficient conditions for the inverse eigenvalue problem of such matrices to be solvable were given. We present some alternative conditions to those given in the aforementioned paper for normal skew *J*-Hamiltonian matrices. By using Moore–Penrose generalized inverse and generalized singular value decomposition, the necessary and sufficient conditions of its solvability are obtained and a solvable general representation is presented.

## Introduction

In this paper, we mainly discuss the following partially described inverse eigenvalue problem which is considered in linear manifold.

### Problem 1

Given the partial eigeninformation $Y = (y_{1},y_{2},\ldots,y_{m}) \in {\mathbb{C}}^{n\times m}$ and $\Lambda= \operatorname{diag}(\lambda_{1},\lambda_{2}, \ldots,\lambda_{m}) \in {\mathbb{C}}^{m\times m}$, consider the set

$$\mathcal{M}(Y,\Lambda):= \{A\in\Omega| AY = Y\Lambda\} $$

of matrices *A* maintaining the eigeninformation, where Ω is the set of certain *n*-by-*n* structured matrices.

The above problem usually appears in the design and modification of mass-spring systems, dynamic structures, Hopfield neural networks, vibration in mechanic, civil engineering, and aviation [2–4]. Furthermore, the inverse eigenvalue problems involving Hamiltonian matrices have drawn considerable interest. For example, Zhang et al. [5] solved the inverse eigenvalue problem of Hermitian and generalized Hamiltonian matrices. Then Bai [6] settled the case of Hermitian and generalized skew-Hamiltonian matrices. Xie et al. [7] resolved the case of symmetric skew-Hamiltonian matrices. Qian and Tan [8] also considered the cases of Hermitian and generalized Hamiltonian/skew-Hamiltonian matrices from different perspectives. But the Hamiltonian matrices they considered are the special cases of the following normal *J*-Hamiltonian matrices and normal skew *J*-Hamiltonian matrices.

In the following, let $I_{n}$ be the $n\times n$ identity matrix.

### Definition 1

([1])

Given a normal matrix $J \in{\mathbb {R}}^{n\times n}$ with $J^{2}=-I_{n}$. *A* matrix $A \in{\mathbb {C}}^{n\times n}$ is referred to as normal *J*-Hamiltonian if and only if ${(AJ)}^{*}=AJ$ and $AA^{*}=A^{*}A$.

### Definition 2

Let $J \in{\mathbb{R}}^{n\times n}$ be a normal matrix such that $J^{2}=-I_{n}$. *A* matrix $A \in{\mathbb{C}}^{n\times n}$ is called normal skew *J*-Hamiltonian if and only if ${(AJ)}^{*}=-AJ$ and $AA^{*}=A^{*}A$. The set of all $n\times n$ normal skew *J*-Hamiltonian matrices is denoted by ${\mathcal{NS}}^{n\times n}(J)$.

In the above definitions, $A^{*}$ signifies the conjugate transpose of a matrix $A \in{\mathbb{C}}^{n\times n}$. It is obvious that *J* is a real orthogonal skew-symmetric matrix, i.e., $J=-J^{T}=-J^{-1}$. This indicates that $n=2k$, $k\in\mathbb{N}$. The above Hamiltonian matrices are also of importance in several engineering areas such as optimal quadratic linear control, $H_{\infty}$ optimization, and the related problem of solving Riccati algebraic equations [9].

Recently, Gigola et al. [1] solved Problem 1 for normal *J*-Hamiltonian matrices. In this paper, we present a set of alternative conditions assuring the solvability of the problem that involves skew normal *J*-Hamiltonian matrices. In order to present more simple conditions to be verified, we mainly use Sun’s [10] and Penrose’s [11] results and the generalized singular value decomposition to solve Problem 1 when the set $\Omega={\mathcal{NS}}^{n\times n}(J)$. A similar technique may be used to solve the inverse eigenvalue problem for normal J-Hamiltonian matrices.

## Preliminaries

Throughout this paper, we denote by $\operatorname{rank}(A)$ and $A^{\dagger}$ the rank and Moore–Penrose generalized inverse of a matrix $A \in{\mathbb {C}}^{n\times m}$, respectively. $I_{n}$, **0**, and $\mathrm {i}=\sqrt{-1}$ respectively signify the identity matrix of order *n*, a zero matrix or a vector with appropriate size, and the imaginary unit. Moreover, for any matrix $A \in{\mathbb{C}}^{n\times m}$, ${\mathscr {R}}_{A}=I_{n}-AA^{\dagger}$ and ${\mathscr{L}}_{A}=I_{m}-A^{\dagger}A$ signify specified orthogonal projectors.

Firstly, we consider the structure of the normal skew *J*-Hamiltonian matrices.

### Lemma 1

*Let* $J \in{\mathbb{R}}^{n\times n}$ *be a normal matrix such that* $J^{2}=-I_{n}$. *Then a matrix* $A \in{\mathbb{C}}^{n\times n}$ *is normal skew* *J*-*Hamiltonian if and only if*

2.1 $$ A=U\left [ \textstyle\begin{array}{c@{\quad}c} A_{11} & A_{12}\\ -A^{*}_{12} & A_{22} \end{array}\displaystyle \right ]U^{*}, $$

*where* $n=2k$, $k\in\mathbb{N}$, $A_{11}=A^{*}_{11}\in{\mathbb{C}}^{k\times k}$, $A_{22}=A^{*}_{22}\in{\mathbb{C}}^{k\times k}$, $A_{11}A_{12}=A_{12}A_{22}$, *and* $U \in{\mathbb{C}}^{n\times n}$ *is a unitary matrix such that*

2.2 $$ U^{*}JU=\left [ \textstyle\begin{array}{c@{\quad}c} \mathrm{i}I_{k} & \bf{0}\\ \bf{0} & -\mathrm{i}I_{k} \end{array}\displaystyle \right ]. $$

### Proof

Because $J \in{\mathbb{R}}^{n\times n}$ is a normal matrix and $J^{2}=-I_{n}$, then *J* is a real orthogonal skew-symmetric matrix. Therefore, there exists a unitary matrix $U \in{\mathbb{C}}^{n\times n}$ such that (2.2) holds, where $n=2k$, $k\in\mathbb{N}$.

Then partition $U^{*}AU$ conforms with (2.2) as

2.3 $$ U^{*}AU=\left [ \textstyle\begin{array}{c@{\quad}c} A_{11} & A_{12}\\ A_{21} & A_{22} \end{array}\displaystyle \right ]. $$

From Definition 2, we know that $JAJ=-A^{*}$. It follows that

$$\left [ \textstyle\begin{array}{c@{\quad}c} -A_{11} & A_{12}\\ A_{21} & -A_{22} \end{array}\displaystyle \right ]=-\left [ \textstyle\begin{array}{c@{\quad}c} A^{*}_{11} & A^{*}_{21}\\ A^{*}_{12} & A^{*}_{22} \end{array}\displaystyle \right ]. $$

Thus we have

2.4 $$ A_{11}=A^{*}_{11},\qquad A_{22}=A^{*}_{22},\qquad A_{12}=-A^{*}_{21}. $$

Because $AA^{*}=A^{*}A$, then from (2.3) and (2.4) we have $A_{11}A_{12}=A_{12}A_{22}$. Therefore, (2.1) holds. □

Then we introduce the following results to solve Problem 1 later on.

### Lemma 2

(Sun [10])

*Let* $A_{1},B_{1}\in{\mathbb {C}}^{n\times m}$ *be given*. *The linear matrix equation* $X_{1}A_{1}=B_{1}$ *has a Hermitian solution* $X_{1}\in{\mathbb{C}}^{n\times n}$ *if and only if*

$$B_{1}{\mathscr{L}}_{A_{1}}=0,\qquad A^{*}_{1}B_{1}=B^{*}_{1}A_{1}. $$

*In this case*, *the general solution can be expressed as*

$$X_{1}=B_{1}A_{1}^{\dagger}+ { \bigl(B_{1}A_{1}^{\dagger}\bigr)}^{*} - \frac{1}{2}{\bigl(A_{1}^{\dagger}\bigr)}^{*} \bigl(A^{*}_{1}B_{1} + B^{*}_{1}A_{1} \bigr)A_{1}^{\dagger}+ {\mathscr{R}}_{A_{1}}R_{1}{ \mathscr {R}}_{A_{1}}, $$

*where* $R_{1}\in{\mathbb{C}}^{n\times n}$ *is an arbitrary Hermitian matrix*.

In this lemma, the general solution can be also expressed as

$$X_{1}=B_{1}A_{1}^{\dagger}+ { \bigl(B_{1}A_{1}^{\dagger}\bigr)}^{*}{ \mathscr{R}}_{A_{1}}+{\mathscr {R}}_{A_{1}}R_{1}{ \mathscr{R}}_{A_{1}}. $$

The following lemma is taken from [11], see Corollary 2 in [11].

### Lemma 3

(Penrose [11])

*Let* $A_{2}\in{\mathbb {C}}^{n\times m}$, $C_{2}\in{\mathbb{C}}^{n\times p}$, $B_{2}\in{\mathbb {C}}^{p\times q}$, *and* $D_{2}\in{\mathbb{C}}^{m\times q}$ *be given*. *The pair of matrix equations* $A_{2}X_{2}=C_{2}$, $X_{2}B_{2}=D_{2}$ *has a solution* $X_{2}\in{\mathbb{C}}^{m\times p}$ *if and only if*

$${\mathscr{R}}_{A_{2}}C_{2}=0,\qquad D_{2}{ \mathscr{L}}_{B_{2}}=0,\qquad A_{2}D_{2}=C_{2}B_{2}. $$

*Moreover*, *the general solution can be expressed as*

$$X_{2}=A_{2}^{\dagger}C_{2} + { \mathscr{L}}_{A_{2}}D_{2}B_{2}^{\dagger}+ { \mathscr {L}}_{A_{2}}R_{2}{\mathscr{R}}_{B_{2}}, $$

*where* $R_{2}\in{\mathbb{C}}^{m\times p}$ *is an arbitrary matrix*.

## Solvability conditions and general solution of Problem 1

Given a normal matrix $J \in{\mathbb{R}}^{n\times n}$ with $J^{2}=-I_{n}$, let $Y \in{\mathbb{C}}^{n\times m}$ and $\Lambda\in {\mathbb{C}}^{m\times m}$ be given in Problem 1. In order to solve this problem for the case of normal skew *J*-Hamiltonian matrices, we need to obtain the normal skew *J*-Hamiltonian solution of the linear matrix equation

3.1 $$ AY=Y\Lambda. $$

If equation (3.1) is consistent, then the set $\mathcal {M}(Y,\Lambda)$ is nonempty. By Lemma 1, equation (3.1) is equivalent to the following:

3.2 $$ \left [ \textstyle\begin{array}{c@{\quad}c} A_{11} & A_{12}\\ -A^{*}_{12} & A_{22} \end{array}\displaystyle \right ]U^{*}Y=U^{*}Y \Lambda. $$

Let

3.3 $$ U^{*}Y=\left [ \textstyle\begin{array}{c} Y_{1} \\ Y_{2} \end{array}\displaystyle \right ],\quad Y_{1} \in{\mathbb{C}}^{k\times m}, Y_{2} \in{\mathbb {C}}^{k\times m}. $$

Then (3.2) can be rewritten as follows:

3.4 $$ \left\{ \textstyle\begin{array}{l}A_{11}Y_{1}+A_{12}Y_{2} = Y_{1}\Lambda,\\ -A^{*}_{12}Y_{1}+A_{22}Y_{2} = Y_{2}\Lambda. \end{array}\displaystyle \right.$$

Thus we have

$$ \left\{ \textstyle\begin{array}{l} A_{12}Y_{2} = Y_{1}\Lambda-A_{11}Y_{1},\\ Y^{*}_{1}A_{12} = Y^{*}_{2}A_{22}-\Lambda^{*}Y^{*}_{2}. \end{array}\displaystyle \right. $$

By Lemma 3, the above system of matrix equations has a solution $A_{12} \in{\mathbb{C}}^{k\times k}$ if and only if

3.5 $$ \left\{ \textstyle\begin{array}{l} A_{11}Y_{1}{\mathscr{L}}_{Y_{2}}=Y_{1}\Lambda{\mathscr{L}}_{Y_{2}},\\ A_{22}Y_{2}{\mathscr{L}}_{Y_{1}}=Y_{2}\Lambda{\mathscr{L}}_{Y_{1}}, \quad\mbox{where } {\mathscr{L}}_{Y_{1}}=I_{k}-Y_{1}Y_{1}^{*},\\ Y^{*}_{1}A_{11}Y_{1}+Y^{*}_{2}A_{22}Y_{2}=Y^{*}_{1}Y_{1}\Lambda+\Lambda^{*}Y^{*}_{2}Y_{2}. \end{array}\displaystyle \right. $$

Then, by Lemma 2, the first equation in (3.5) has a Hermitian solution $A_{11}\in{\mathbb{C}}^{k\times k}$ if and only if

3.6 $$ Y_{1}\Lambda{\mathscr{L}}_{Y_{2}}{ \mathscr{L}}_{Y_{1}{\mathscr{L}}_{Y_{2}}}=0,\qquad {\mathscr{L}}_{Y_{2}}\bigl(Y^{*}_{1}Y_{1} \Lambda-\Lambda^{*}Y^{*}_{1}Y_{1}\bigr){\mathscr{L}}_{Y_{2}}=0. $$

In this case, the general solution is

3.7 $$ A_{11}=Y_{1}\Lambda{\mathscr{L}}_{Y_{2}}{(Y_{1}{ \mathscr{L}}_{Y_{2}})}^{\dagger}+ {\bigl({\mathscr{L}}_{Y_{2}}Y^{*}_{1} \bigr)}^{\dagger}{\mathscr{L}}_{Y_{2}}\Lambda ^{*}Y^{*}_{1}{ \mathscr{R}}_{Y_{1}{\mathscr{L}}_{Y_{2}}}+ {\mathscr {R}}_{Y_{1}{\mathscr{L}}_{Y_{2}}}S_{1}{ \mathscr{R}}_{Y_{1}{\mathscr{L}}_{Y_{2}}}, $$

where $S_{1}\in{\mathbb{C}}^{k\times k}$ is an arbitrary Hermitian matrix.

Similarly, by Lemma 2, the second equation in (3.5) has a Hermitian solution $A_{22}\in{\mathbb{C}}^{k\times k}$ if and only if

3.8 $$ Y_{2}\Lambda{\mathscr{L}}_{Y_{1}}{ \mathscr{L}}_{Y_{2}{\mathscr{L}}_{Y_{1}}}=0,\qquad {\mathscr{L}}_{Y_{1}}\bigl(Y^{*}_{2}Y_{2} \Lambda-\Lambda^{*}Y^{*}_{2}Y_{2}\bigr){\mathscr{L}}_{Y_{1}}=0. $$

In this case, the general solution is

3.9 $$ A_{22}=Y_{2}\Lambda{\mathscr{L}}_{Y_{1}}{(Y_{2}{ \mathscr{L}}_{Y_{1}})}^{\dagger}+ {\bigl({\mathscr{L}}_{Y_{1}}Y^{*}_{2} \bigr)}^{\dagger}{\mathscr{L}}_{Y_{1}}\Lambda ^{*}Y^{*}_{2}{ \mathscr{R}}_{Y_{2}{\mathscr{L}}_{Y_{1}}}+ {\mathscr {R}}_{Y_{2}{\mathscr{L}}_{Y_{1}}}S_{2}{ \mathscr{R}}_{Y_{2}{\mathscr{L}}_{Y_{1}}}, $$

where $S_{2}\in{\mathbb{C}}^{k\times k}$ is an arbitrary Hermitian matrix. Let

3.10 $$ \begin{aligned}[b]G ={}& Y^{*}_{1}Y_{1}\Lambda+ \Lambda^{*}Y^{*}_{2}Y_{2}-Y^{*}_{1}\bigl[Y_{1} \Lambda{\mathscr {L}}_{Y_{2}}{(Y_{1}{\mathscr{L}}_{Y_{2}})}^{\dagger}+ {\bigl({\mathscr {L}}_{Y_{2}}Y^{*}_{1}\bigr)}^{\dagger}{ \mathscr{L}}_{Y_{2}}\Lambda^{*}Y^{*}_{1}{\mathscr {R}}_{Y_{1}{\mathscr{L}}_{Y_{2}}}\bigr]Y_{1} \\ & -Y^{*}_{2}\bigl[Y_{2}\Lambda{\mathscr{L}}_{Y_{1}}{(Y_{2}{ \mathscr{L}}_{Y_{1}})}^{\dagger}+ {\bigl({\mathscr{L}}_{Y_{1}}Y^{*}_{2} \bigr)}^{\dagger}{\mathscr{L}}_{Y_{1}}\Lambda ^{*}Y^{*}_{2}{ \mathscr{R}}_{Y_{2}{\mathscr{L}}_{Y_{1}}}\bigr]Y_{2}.\end{aligned} $$

From (3.5), (3.7), and (3.9) we know that $G=G^{*}$ is equivalent to

$$Y^{*}_{1}Y_{1}\Lambda+\Lambda^{*}Y^{*}_{2}Y_{2}= \Lambda^{*}Y^{*}_{1}Y_{1}+Y^{*}_{2}Y_{2} \Lambda. $$

Then substituting (3.7) and (3.9) into the third equation in (3.5) yields

3.11 $$ Y^{*}_{1}{\mathscr{R}}_{Y_{1}{\mathscr{L}}_{Y_{2}}}S_{1}{ \mathscr {R}}_{Y_{1}{\mathscr{L}}_{Y_{2}}}Y_{1} +Y^{*}_{2}{ \mathscr{R}}_{Y_{2}{\mathscr{L}}_{Y_{1}}}S_{2}{\mathscr {R}}_{Y_{2}{\mathscr{L}}_{Y_{1}}}Y_{2}=G. $$

Thus we need to obtain a pair of Hermitian solutions $( \widehat{S}_{1}, \widehat{S}_{2})$ of the linear matrix equation (3.11). Firstly, we give the generalized singular value decomposition (GSVD) of the matrix pair $({\mathscr{R}}_{Y_{1}{\mathscr{L}}_{Y_{2}}}Y_{1}, {\mathscr {R}}_{Y_{2}{\mathscr{L}}_{Y_{1}}}Y_{2})$ as follows (see, for example, [12]):

3.12 $$ {\mathscr{R}}_{Y_{1}{\mathscr{L}}_{Y_{2}}}Y_{1}=U_{1} \Pi_{1}M, \qquad{\mathscr {R}}_{Y_{2}{\mathscr{L}}_{Y_{1}}}Y_{2}=U_{2} \Pi_{2}M, $$

where $U_{1}$ and $U_{2}$ are unitary matrices of order *k* and $M \in {\mathbb{C}}^{m\times m}$ is a nonsingular matrix, and

$$\begin{gathered} \Pi_{1}= \textstyle\begin{array}{cl} \left ( \textstyle\begin{array}{c@{\qquad}c@{\qquad}c@{\qquad}c} I_{r_{3}-r_{2}} & \qquad\bf{0} & \qquad\bf{0} & \quad\bf{0}\\ \bf{0} & \qquad\Lambda_{1} & \qquad\bf{0} & \quad\bf{0}\\ \bf{0} & \qquad\bf{0} & \qquad\bf{0} & \quad\bf{0} \end{array}\displaystyle \right ) & \left . \textstyle\begin{array}{l} r_{3}-r_{2} \\ r_{1}+r_{2}-r_{3}\\ k-r_{1} \end{array}\displaystyle \right . \\ \left . \textstyle\begin{array}{@{\quad}c@{\qquad}c@{\quad}c@{\quad}c} r_{3}-r_{2} & r_{1}+r_{2}-r_{3} & r_{3}-r_{1} & m-r_{3} \end{array}\displaystyle \right . & \end{array}\displaystyle , \\\Pi_{2}= \textstyle\begin{array}{cl} \left ( \textstyle\begin{array}{c@{\qquad}c@{\qquad}c@{\qquad}c} \bf{0} & \quad\hspace{6pt}\bf{0} & \quad\bf{0} & \quad\bf{0}\\ \bf{0} & \quad\hspace{6pt}\Lambda_{2} & \quad\bf{0} & \quad\bf{0}\\ \bf{0} & \quad\hspace{6pt}\bf{0} & \quad I_{r_{3}-r_{1}} & \quad\bf{0} \end{array}\displaystyle \right ) & \left . \textstyle\begin{array}{l} k-r_{2} \\ r_{1}+r_{2}-r_{3}\\ r_{3}-r_{1} \end{array}\displaystyle \right . \\ \left . \textstyle\begin{array}{c@{\quad}c@{\quad}c@{\quad}c} r_{3}-r_{2} & r_{1}+r_{2}-r_{3} & r_{3}-r_{1} & \quad m-r_{3} \end{array}\displaystyle \right . & \end{array}\displaystyle \end{gathered} $$

are block matrices with the same column partitioning. In the matrices $\Pi_{1}$ and $\Pi_{2}$,

$$\begin{aligned}& r_{1} = \operatorname{rank}({\mathscr{R}}_{Y_{1}{\mathscr{L}}_{Y_{2}}}Y_{1}),\qquad r_{2} = \operatorname{rank}({\mathscr{R}}_{Y_{2}{\mathscr{L}}_{Y_{1}}}Y_{2}),\\& r_{3} = \operatorname{rank}\bigl(Y^{*}_{1}{\mathscr{R}}_{Y_{1}{\mathscr{L}}_{Y_{2}}},Y^{*}_{2}{ \mathscr {R}}_{Y_{2}{\mathscr{L}}_{Y_{1}}}\bigr), \\& \Lambda_{1} = \operatorname{diag}(\xi_{1},\xi_{2}, \ldots,\xi_{r_{1}+r_{2}-r_{3}}),\quad 1>\xi _{1}\geq\cdots\geq \xi_{r_{1}+r_{2}-r_{3}}>0, \\& \Lambda_{2} = \operatorname{diag}(\eta_{1},\eta_{2}, \ldots,\eta_{r_{1}+r_{2}-r_{3}}),\quad 0< \eta_{1}\leq\cdots\leq \eta_{r_{1}+r_{2}-r_{3}}< 1, \\& \Lambda^{2}_{1}+\Lambda^{2}_{2}=I_{r_{1}+r_{2}-r_{3}}. \end{aligned}$$

We further partition the nonsingular matrix

$$M^{-1}=\left ( \textstyle\begin{array}{c@{\quad}c@{\quad}c@{\quad}c} M_{1} & M_{2} & M_{3} & M_{4}\\ r_{3}-r_{2} & r_{1}+r_{2}-r_{3} & r_{3}-r_{1} & m-r_{3} \end{array}\displaystyle \right ) $$

compatibly with the block column partitioning of $\Pi_{1}$ or $\Pi_{2}$. Denote

3.13 $$ {\bigl(M^{*}\bigr)}^{-1}GM^{-1}=(G_{ij})_{4\times4}\quad \mbox{with } G_{ij}=M^{*}_{i}GM_{j}, i,j=1,2,3,4. $$

Then substitute (3.12) into (3.11). By [13, Theorem 3.1] we obtain that equation (3.11) is consistent if and only if

3.14 $$ \begin{gathered} Y^{*}_{1}Y_{1}\Lambda+\Lambda^{*}Y^{*}_{2}Y_{2}= \Lambda^{*}Y^{*}_{1}Y_{1}+Y^{*}_{2}Y_{2} \Lambda,\\ G_{13}=0,\qquad G_{14}=0,\qquad G_{24}=0,\qquad G_{34}=0,\qquad G_{44}=0.\end{gathered} $$

Moreover, its general solution can be expressed as

3.15 $$\begin{aligned}& \widehat{S}_{1}=U_{1}\left [ \textstyle\begin{array}{c@{\quad}c@{\quad}c} G_{11} & G_{12}\Lambda_{1}^{-1} & X_{13}\\ \Lambda_{1}^{-1}G^{*}_{12} & \Lambda_{1}^{-1}(G_{22}-\Lambda_{2}Y_{22}\Lambda _{2})\Lambda_{1}^{-1} & X_{23}\\ X^{*}_{13} & X^{*}_{23} & X_{33} \end{array}\displaystyle \right ]U^{*}_{1}, \end{aligned}$$

3.16 $$\begin{aligned}& \widehat{S}_{2}=U_{2}\left [ \textstyle\begin{array}{c@{\quad}c@{\quad}c} Y_{11} & Y_{12} & Y_{13}\\ Y^{*}_{12} & Y_{22} & \Lambda_{2}^{-1}G_{23}\\ Y^{*}_{13} & G^{*}_{23}\Lambda_{2}^{-1} & G_{33} \end{array}\displaystyle \right ]U^{*}_{2}, \end{aligned}$$

where $X_{33}$, $Y_{11}$, and $Y_{22}$ are arbitrary Hermitian matrices, $X_{13}$, $X_{23}$, $Y_{12}$, and $Y_{13}$ are arbitrary matrices.

Then substituting (3.15) and (3.16) into (3.7) and (3.9) yields

3.17 $$ \left\{ \textstyle\begin{array}{l} A_{11}=Y_{1}\Lambda{\mathscr{L}}_{Y_{2}}{(Y_{1}{\mathscr{L}}_{Y_{2}})}^{\dagger}+{({\mathscr{L}}_{Y_{2}}Y^{*}_{1})}^{\dagger}{\mathscr{L}}_{Y_{2}} \Lambda^{*}Y^{*}_{1}{\mathscr{R}}_{Y_{1}{\mathscr{L}}_{Y_{2}}} +{\mathscr{R}}_{Y_{1}{\mathscr{L}}_{Y_{2}}}\widehat{S}_{1}{\mathscr {R}}_{Y_{1}{\mathscr{L}}_{Y_{2}}},\\ A_{22}=Y_{2}\Lambda{\mathscr{L}}_{Y_{1}}{(Y_{2}{\mathscr{L}}_{Y_{1}})}^{\dagger}+{({\mathscr{L}}_{Y_{1}}Y^{*}_{2})}^{\dagger}{\mathscr{L}}_{Y_{1}} \Lambda^{*}Y^{*}_{2}{\mathscr{R}}_{Y_{2}{\mathscr{L}}_{Y_{1}}} +{\mathscr{R}}_{Y_{2}{\mathscr{L}}_{Y_{1}}}\widehat{S}_{2}{\mathscr {R}}_{Y_{2}{\mathscr{L}}_{Y_{1}}}. \end{array}\displaystyle \right. $$

From (3.4), (3.17), and Lemma 3, we get

3.18 $$ \begin{aligned}[b] A_{12} ={}& {\bigl(Y^{*}_{1} \bigr)}^{\dagger}\bigl(Y^{*}_{2}A_{22}- \Lambda^{*}Y^{*}_{2}\bigr)+{\mathscr {R}}_{Y_{1}}(Y_{1} \Lambda-A_{11}Y_{1}){\bigl(Y^{*}_{2} \bigr)}^{\dagger}+ {\mathscr {R}}_{Y_{1}}R{\mathscr{R}}_{Y_{2}} \\ ={}& {\bigl(Y^{\dagger}_{1}\bigr)}^{*}Y^{*}_{2}Y_{2} \Lambda{\mathscr{L}}_{Y_{1}}{(Y_{2}{\mathscr {L}}_{Y_{1}})}^{\dagger}+ {\bigl(Y^{\dagger}_{1}\bigr)}^{*}Y^{*}_{2} {\bigl({\mathscr {L}}_{Y_{1}}Y_{2}^{*}\bigr)}^{\dagger}{\mathscr{L}}_{Y_{1}} \Lambda^{*}Y^{*}_{2}{\mathscr {R}}_{Y_{2}{\mathscr{L}}_{Y_{1}}} \\ &-{\mathscr{R}}_{Y_{1}}{\bigl({\mathscr{L}}_{Y_{2}}Y^{*}_{1} \bigr)}^{\dagger}{\mathscr {L}}_{Y_{2}}\Lambda^{*}Y^{*}_{1}{ \mathscr{R}}_{Y_{1}{\mathscr {L}}_{Y_{2}}}Y_{1}{Y_{2}}^{\dagger}-{ \bigl(Y^{\dagger}_{1}\bigr)}^{*}\Lambda^{*}Y^{*}_{2} \\ &+{\bigl(Y^{\dagger}_{1}\bigr)}^{*}Y^{*}_{2}{ \mathscr{R}}_{Y_{2}{\mathscr{L}}_{Y_{1}}}\widehat {S}_{2}{\mathscr{R}}_{Y_{2}{\mathscr{L}}_{Y_{1}}}- {\mathscr{R}}_{Y_{1}}{\mathscr{R}}_{Y_{1}{\mathscr{L}}_{Y_{2}}}\widehat {S}_{1}{\mathscr{R}}_{Y_{1}{\mathscr{L}}_{Y_{2}}}Y_{1}{Y_{2}}^{\dagger}\\ &+{\mathscr{R}}_{Y_{1}}R{\mathscr{R}}_{Y_{2}} ,\end{aligned} $$

where $R \in{\mathbb{C}}^{k\times k}$ is an arbitrary matrix.

Based on the above discussion, we can conclude the following result to solve Problem 1.

### Theorem 1

*Given* $Y \in{\mathbb{C}}^{n\times m}$ *and* $\Lambda\in {\mathbb{C}}^{m\times m}$ *as described in Problem* 1. *Let* $U^{*}Y$, *G*, *the GSVD of the matrix pair* $({\mathscr{R}}_{Y_{1}{\mathscr {L}}_{Y_{2}}}Y_{1}, {\mathscr{R}}_{Y_{2}{\mathscr{L}}_{Y_{1}}}Y_{2})$ *and* ${(M^{*})}^{-1}G{M}^{-1}$ *be given by* (3.3), (3.10), (3.12), *and* (3.13), *respectively*. *Then Problem* 1 *is solvable*(*i*.*e*., $\mathcal{M}(Y,\Lambda)\neq\emptyset$) *in the set* ${\mathcal{NS}}^{n\times n}(J)$ *if and only if conditions* (3.6), (3.8), (3.14), *and* $A_{11}A_{12}=A_{12}A_{22}$ *hold*. *Moreover*, *in this case*, *the general solution can be expressed as*

$$A=U\left [ \textstyle\begin{array}{c@{\quad}c} A_{11} & A_{12}\\ -A^{*}_{12} & A_{22} \end{array}\displaystyle \right ]U^{*}, $$

*where* $A_{11}$, $A_{22}$, *and* $A_{12}$ *are given by* (3.17) *and* (3.18), *respectively*. *In the matrices* $A_{11}$, $A_{22}$, *and* $A_{12}$, $\widehat{S}_{1}$ *and* $\widehat{S}_{2}$ *are described in* (3.15) *and* (3.16), *respectively*, *where* $X_{33}$, $Y_{11}$, *and* $Y_{22}$ *are arbitrary Hermitian matrix blocks*, $X_{13}$, $X_{23}$, $Y_{12}$, $Y_{13}$, *and* *R* *are arbitrary matrix blocks*.

## Conclusions

In this paper, we have obtained the necessary and sufficient conditions of the inverse eigenvalue problem for normal skew *J*-Hamiltonian matrices. Furthermore, a solvable general representation is presented. We can also use the same method to solve the inverse eigenvalue problem for a normal *J*-Hamiltonian matrix.
